# Assessing the feasibility of neonatal chest MRI for bronchopulmonary dysplasia using a standard 1.5-Tesla scanner

**DOI:** 10.1007/s00330-026-12452-4

**Published:** 2026-03-24

**Authors:** Jantine J. Wisse, Bernadette B. L. J. Elders, Merlijn Bonte, Piotr A. Wielopolski, André A. Kroon, Harm A. W. M. Tiddens, Liesbeth Duijts, Mariëlle W. H. Pijnenburg, Irwin K. M. Reiss, Pierluigi Ciet

**Affiliations:** 1 Division of Neonatology, Department of Neonatal and Pediatric Intensive Care, Erasmus MC, University Medical Center Rotterdam, Rotterdam, The Netherlands; 2 Department of Adult Intensive Care, Erasmus MC, University Medical Center Rotterdam, Rotterdam, The Netherlands; 3 Division of Respiratory Medicine and Allergology, Department of Pediatrics, Erasmus MC, University Medical Center Rotterdam, Rotterdam, The Netherlands; 4 Department of Radiology and Nuclear Medicine, Erasmus Medical Centre—Sophia Children’s Hospital, Rotterdam, The Netherlands; 5 Thirona B.V, Nijmegen, The Netherlands; 6 Medical University Eppendorf, Medical Children’s Hospital, Divison of Neonatology and Pediatric Intensive Care, Hamburg, Germany; 7 Department of Radiology, Policlinic University Hospital, Cagliari, Italy

**Keywords:** Bronchopulmonary dysplasia, CT, Imaging, MRI, Neonatology

## Abstract

**Objective:**

Accurate imaging is essential for assessing structural lung abnormalities in children with bronchopulmonary dysplasia (BPD). Chest MRI offers a radiation-free alternative to CT and enables silent scanning. This study aimed to develop and evaluate a chest MRI protocol for BPD using a standard 1.5-Tesla (T) MRI scanner with a dedicated neonatal chest coil.

**Materials and methods:**

In this prospective pilot study, infants underwent feed-and-swaddle chest MRI at ~40 weeks postmenstrual age, and chest CT at six months corrected age. The MRI protocol included free-breathing axial T2-weighted (T2-W) fast spin echo (PROPELLER) and axial proton density-weighted (PD-W) gradient zero echo time (ZTE) sequences. Scans were assessed for image quality, and quantified for normal, hypo-/hyperintense lung tissue and bronchopathy. MRI and CT scores were correlated using Pearson’s or Spearman’s coefficients, based on data distribution.

**Results:**

Eight infants participated (seven preterm < 28 weeks’ gestation with severe BPD; one term neonate with asphyxia). T2-w PROPELLER provided superior soft tissue contrast and fewer artefacts than ZTE. ZTE enabled silent scanning and better visualisation of hypointense structures. Quantitative scores were comparable between PROPELLER and ZTE sequences. Significant correlations were found between MRI and CT scores for normal and hyperintense lung tissue (*p* < 0.05; *r* = 0.84–0.96), but not for hypointense regions and bronchopathy.

**Conclusion:**

We developed a feasible and safe chest MRI protocol for imaging severe BPD-related lung abnormalities in neonates using a standard 1.5-T system. While technically promising, MRI is not yet clinically equivalent to CT. Further validation is needed to define its potential role in BPD assessment.

**Key Points:**

***Question*** *Can a standard 1.5 T chest MRI protocol performed without anaesthesia reliably image lung abnormalities in neonates with BPD?*

***Findings**** T2-W PROPELLER sequence provided superior image quality and fewer artefacts compared to the ZTE, especially in depicting fissures and bronchi*.

***Clinical relevance**** Our BPD-MRI protocol enables safe, radiation-free lung imaging in neonates with severe BPD without requiring anaesthesia. It offers a technically feasible alternative to CT and may support early structural assessment before discharge in routine clinical care*.

**Graphical Abstract:**

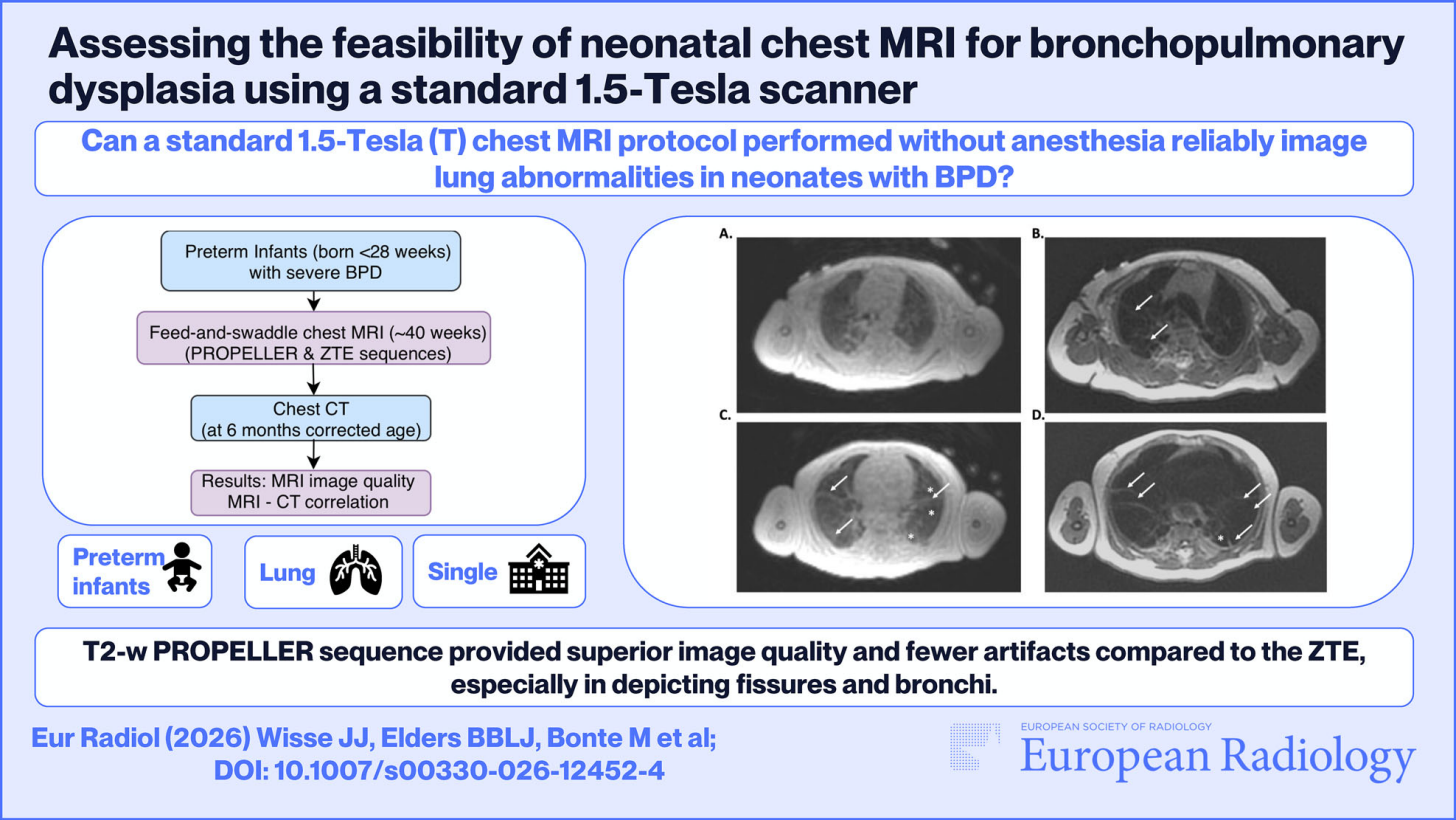

## Introduction

Bronchopulmonary dysplasia (BPD) is the most common chronic lung disease in preterm infants [1]. BPD results from disrupted alveolar and vascular development due to premature birth, leading to ventilation and perfusion abnormalities, inflammation and structural lung abnormalities. BPD severity is currently classified using the National Institute of Health criteria, which are based on the need for supplemental oxygen at 36 weeks postmenstrual age (PMA) [2, 3]. Despite improvements in perinatal care [4, 5], structural lung abnormalities, such as alveolar simplification and impaired vascular development, remain prevalent. A validated imaging method for accurately assessing impaired lung structure is still lacking, both in the neonatal period and later in life. Currently, structural lung imaging is recommended only for infants with severe BPD [6], but the specific lung structural abnormalities that predict long-term respiratory morbidity remain unclear. Some children primarily suffer from parenchymal diseases, while others show large airway or pulmonary vascular involvement, each presenting distinct implications for treatment and prognosis [7]. This heterogeneity highlights the need for improved phenotyping to support personalised therapeutic strategies.

Recent technical developments in Magnetic Resonance Imaging (MRI) have made it feasible to image various paediatric lung diseases [8, 9]. MRI is particularly attractive in neonates because, unlike computed tomography (CT), it avoids ionising radiation, which is especially important in this vulnerable population [10]. Moreover, MRI allows for dynamic imaging of the central airways and lungs [11, 12], making it a promising modality for functional lung assessment in BPD.

Several studies have shown the feasibility of neonatal chest MRI to assess lung structure in BPD and its correlation with short-term clinical outcomes [13–16]. However, these studies relied on a modified, small-footprint MRI system originally designed for adult orthopaedic imaging, which is not widely available. For MRI to become a standard tool in clinical practice for BPD monitoring, the imaging protocol needs to be reproducible on conventional MRI systems. Recent studies assessed structural abnormalities in school-aged BPD children and ventilation/perfusion defects in infants with BPD using standard MRI systems [17]. In the current Ventilation, Inflammation, perfusion and structure in Neonatal Lung patients (VINyL) study, we aimed to develop and evaluate a chest MRI protocol for neonates with BPD, using a standard 1.5-Tesla (T) MRI system and a dedicated neonatal chest coil. This feasibility study presents findings from our pilot study involving severe BPD neonates and non-premature patients without pulmonary or cardiac comorbidities.

## Methods

This prospective pilot study was approved by the local medical ethics committee (MEC-2019-0378). Written informed consent was obtained from parents or legal caregivers.

### Protocol development

**Fig. 1:**
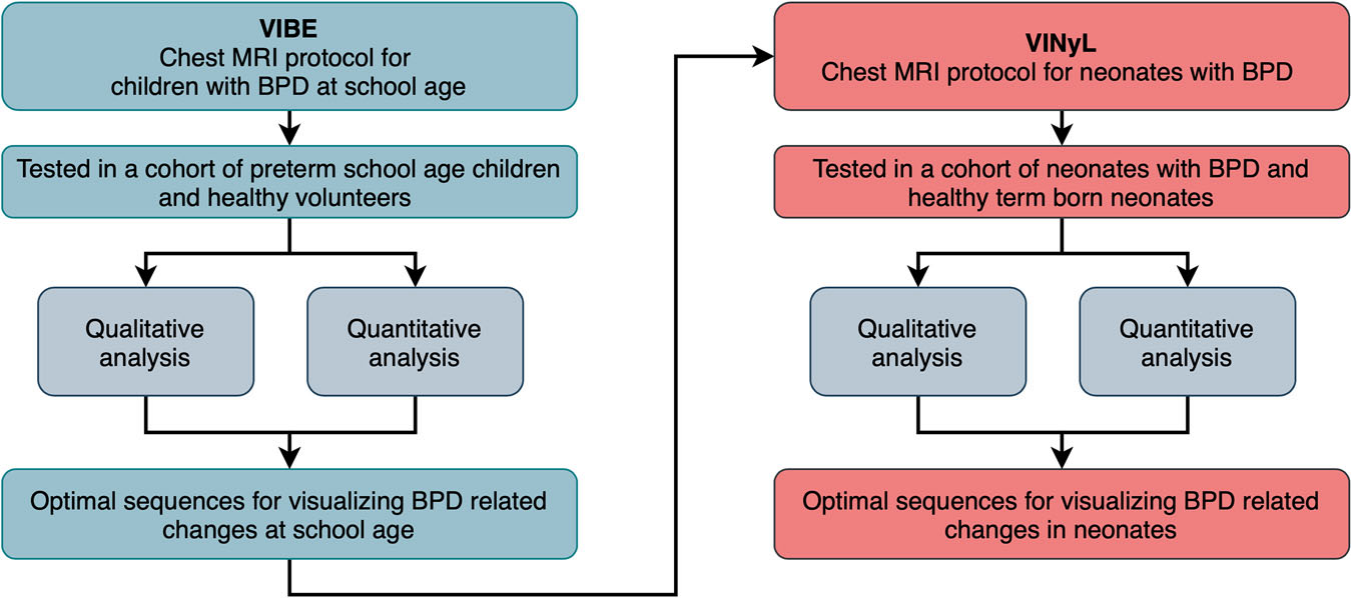
Flowchart of the development of an optimal chest MRI protocol for neonates with BPD in the VINyL study. BPD; bronchopulmonary dysplasia, MRI; magnetic resonance imaging, VIBE; ventilation, inflammation, perfusion and structure in BPD at school age, VINyL; ventilation, inflammation, perfusion and structure in neonates with lung disease

Figure 1 shows the development and testing of our neonatal chest MRI protocol. Sequence selection was guided by a literature review to identify optimal MRI sequences for imaging BPD-related lung abnormalities, and a prior validated protocol used in school-aged children with BPD [17, 18]. The neonatal protocol included a T2-weighted (T2-w) periodically rotated overlapping parallel lines with enhanced reconstruction (PROPELLER) sequence to image hyperintense regions and a proton density-weighted (PD-w) zero echo time (ZTE) sequence for visualising hypointense regions and airway abnormalities. Imaging was implemented on a 1.5-T scanner (*SIGNA Explorer, GE Healthcare)* with a dedicated neonatal chest coil (*LMT Medical Systems GmbH)* and tested at multiple spatial resolutions to optimise image quality and scan time (Table 1).

**Table 1:** Chest BPD-MRI protocol as used in the VINyL study

Sequence	T2-w PROPELLER	PD-w ZTE
Acquisition plane	Axial	Axial
TR/TE (ms)	7500/54	(540–920)/(0.016–0.02)
Flip angle (°)	90/120	2
RF	Selective	Non-selective
In-plane matrix	192 × 192	(140–180)−(140-180)
k-space trajectory	Blades	Radial
In-plane FOV	23	18
Actual voxel resolution (mm^3^)	1.2 × 1.2 × 1.2	(1.0–1.3) × (1.0–1.3) × (1.0–1.3)
Slices	64	100
Slice thickness	1.2	(1.0–1.3)
Receiver bandwidth (kHz)	50.0	31.3
Parallel imaging	3.0	None
Number of averages	6	4
Number of phases	-	-
Number of spokes per segment	16	512
Physiological triggering	None free breathing	None free breathing
Scan time	±9 min	±4 min

ZTE scans were performed at multiple spatial resolutions, the range of the sequences is provided in the table

*FOV* field of view, *PROPELLER* periodically overlapping parallel lines with enhanced reconstruction, *PD-w* proton density weighted, *RF* radio frequency, *T2-W* T2 weighted, *TE* echo time, *TR* repetition time, *ZTE* zero echo time

### Protocol testing

The protocol was tested in neonates with severe BPD and one term control without pulmonary or cardiac comorbidities. Preterm infants (< 28 weeks of gestation) with severe BPD, defined by National Institute of Health criteria at 36 weeks of PMA, and a clinically stable term control (> 37 weeks) were recruited from the neonatal intensive care unit between January 2021 and September 2023. Exclusion criteria for both groups included MRI contraindication (e.g. clinical instability, mechanical ventilation) and congenital cardiovascular or pulmonary abnormalities that could interfere with imaging.

Chest MRI was performed without sedation using a feed-and-swaddle technique. Infants were fed, swaddled and immobilised using a vacuum mattress and soothed with a pacifier and sucrose if needed. The protocol lasted up to 60 min or until signs of discomfort were observed, as determined by the healthcare provider overseeing the MRI examination.

### Qualitative analysis

Two scorers independently assessed image quality on all sequences using the method described by Bae et al (Table 2) [19]. This assessment focused on the presence of artefacts, such as blurring, wrapping, streaking and low signal-to-noise (SNR).

**Table 2:** Qualitative MRI scoring method [19]

Depiction of fissures	1. Unacceptable (invisible interlobar fissure) 2. Fair (blurred interlobar fissure) 3. Good (visible interlobar fissure)
Depiction of intrapulmonary vessels	1. Unacceptable (invisible peripheral pulmonary vessels) 2. Poor (barely visible peripheral pulmonary vessels) 3. Fair (visible peripheral pulmonary vessels) 4. Good (visible peripheral pulmonary vessels with clear margin) 5. Excellent (visible peripheral pulmonary vessels with clear margin)
Depiction of bronchi	1. Unacceptable (indistinguishable lobar bronchial walls) 2. Poor (visible lobar bronchial walls with < 10 visible segmental bronchial walls) 3. Fair (visible lobar bronchial walls with > 10 visible segmental bronchial walls) 4. Good (visible lobar bronchial walls with > 10 visible segmental bronchial walls, with few visible sub/segmental bronchial walls) 5. Excellent (visible sub-subsegmental bronchial walls)
Image noise/artefacts	1. Unacceptable 2. Above average noise/artefacts 3. Average and acceptable 4. Less than average noise/artefacts 5. Minimum of no noise/artefacts
Overall acceptability	1. Unacceptable 2. Suboptimal 3. Satisfactory 4. Above average 5. Superior

### Quantitative analysis

**Fig. 2:**
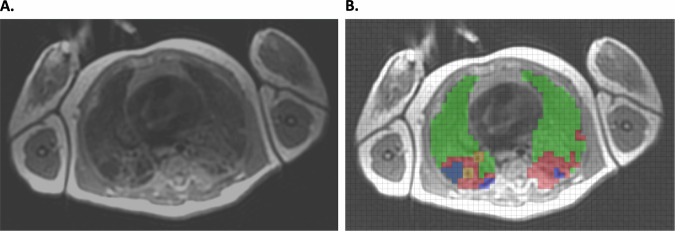
axial T2- weighted PROPELLER sequence of a BPD patient (**A**) with corresponding grid-based scoring system showing different BPD-related lung abnormalities: hypointense regions (blue), hyperintense regions (red), bronchopathy (orange) and normal lung tissue (green) (**B**)

Quantitative analysis involved calculating the volume and percentage of BPD-related lung abnormalities on MRI using a morphometry-based grid-based scoring system, which was adapted from the Perth-Rotterdam Annotated Grid Morphometric Analysis (PRAGMA-BPD) scoring system: the bronchopulMonary rottErdam mRi morpholoGy scorE (MERGE) scoring system [17, 20–22]. Despite scoring methods being comparable, PRAGMA-BPD is specifically designed for CT imaging, while the MERGE score was adapted for MRI. For instance, bronchial wall thickening is scored as part of ‘bronchopathy’ in the MERGE score due to limitations in MR spatial resolution. A grid was placed over ten equidistant axial MR images with the grid-size adapted to the thoracic width. Grids were hierarchically colour-coded to annotate BPD-related abnormalities, including hypointense regions (e.g. mosaic perfusion, emphysema, trapped air, bullae plural opacities); bronchopathy (e.g. bronchiectasis, airway wall thickening) and normal lung tissue. Abnormalities were scored only if they occupied at least 50% of the grid cell. The scoring system prioritised hypointense regions based on prior studies showing that these abnormalities correlated strongly with clinical and lung function outcomes, followed by hyperintense regions (e.g. consolidations, atelectasis and linear/subpleural abnormalities) [21]. Data were presented as a percentage of total lung volume. A semi-automated, in-house developed software was used for MERGE analysis. Figure 2 illustrates the grid-based scoring system on a neonatal MR image.

### Quantitative CT-analysis

As part of routine clinical care, infants with severe BPD underwent chest CT at approximately 6 months corrected age. CT scans were performed using a standard free-breathing protocol without anaesthesia using the Somaton Drive scanner (SIEMENS Healthineers) [23]. CT scans were analysed using the PRAGMA-BPD scoring method [21, 22]. Since CT is the current gold standard for assessing structural lung changes in children with BPD [22], the quantitative MRI scores were compared with the PRAGMA-BPD scores from the CT scans. MRI scans were performed at 40 weeks PMA, whereas the CT scans were conducted at 6 months of corrected age according to standard BPD follow-up, introducing a time gap (~ 214 days) between the examinations.

### MERGE and PRAGMA-BPD scoring

MERGE scoring was done by a trained researcher (B.E.) with over six years of experience in chest MRI. To assess intra-observer variability, B.E re-scored all MRI scans after a four-week interval to prevent recall bias. Inter-observer variation was assessed by a second researcher (M.B.) with over seven years of experience in PRAGMA-BPD scoring, who independently scored all MRI and CT scans. All scoring was blinded to patient identifiers and performed in random order. MRI sequences were reviewed in a fixed order: first ZTE, followed by PROPELLER. Inter- and intra-observer variability was assessed using Bland–Altman analysis based on the percentual differences in assigned scores. The mean bias obtained from this analysis was used as an indicator of variability.

### Statistics

Data analysis was done using R-statistics (version 2022.07.2, RStudio, Posit Software). Descriptive statistics are presented as mean with standard deviation (± SD) for normally distributed data and median with interquartile range [IQR] for non-parametric data. The Shapiro–Wilk test was used to assess data normality. Pearson’s and Spearman’s correlation coefficients were calculated to compare the PRAGMA-BPD score from CT-scan with the MERGE score from MRI, based on data distribution. We did not correct for multiple testing.

## Results

### Study population

Seven infants with severe BPD and one term infant were included (Table 3). The median gestational age at birth for infants with severe BPD was 26.4 weeks, with a median birth weight of 803 grams. The median PMA at MRI was 39.9 weeks. The term neonate was born at 39.4 weeks of gestation with a birth weight of 3130 grams and was admitted to the neonatal intensive care unit for perinatal asphyxia due to severe anaemia, requiring non-invasive respiratory support. All participants underwent an MRI scan at a mean PMA of 39.95 (± 2.9) weeks. Six infants with severe BPD underwent CT-scanning at a median age of 46.7 weeks after birth, while the term infant and one BPD patient (not enrolled in the BPD follow-up program) did not have a CT scan.

**Table 3:** Characteristics and baseline measurements of all study participants

	BPD (*n* = 7)	Control (*n* = 1)
Sex (female)	3 (43%)	1 (100%)
Birthweight	790 [734 815]	3130
Weight at MRI (grams)	2545 (842)	3130
Gestational age at birth	26.4 [25.8 26.5]	39.4
Invasive mechanical ventilation (days)	23 (17.3)	-
Supplemental oxygen > 21% (days)	64.7 (23.8)	-
Age at MRI (PMA)	39.95 (2.9)	39.97
Age at CT (days after birth)	320 (20.9)	-

Data presented as median [IQR] or mean ± sd

*PMA* postmenstrual age

### Qualitative analyses

The MRI protocol included an axial T2-w PROPELLER sequence and axial PD-w ZTE sequence with multiple averages to reduce motion artefacts and improve image quality. Based on initial evaluations, the PD-w ZTE sequence with the highest resolution was selected for its superior image quality (Table 1). Both sequences were successfully performed in all patients, achieving a 100% success rate. The number of scan resolutions varied between patients, as some scans were occasionally discontinued due to patient discomfort or because the maximum scan time was reached.

Qualitative scores for the MRI sequences are presented in Table 4. The T2-w PROPELLER sequence provided superior depiction of lung fissures, vessels, and bronchi with fewer artefacts compared to ZTE sequences. ZTE sequences showed artefacts in 6 of 8 patients, primarily low SNR and blurring caused by motion. Despite these artefacts, overall image acceptability ranged from ‘suboptimal’ to ‘satisfactory’ for ZTE sequences and from ‘satisfactory’ to ‘above average’ for PROPELLER sequences.

**Table 4:** Qualitative MRI scoring

	ZTE	PROPELLER
Fissures Vessels Bronchi Artefacts Acceptability	1.5 [1 2] 2 [2 3] 2 [2 2.25] 2 [2 3.5] 2.5 [2 3.25]	2 [2 2] 3 [2.75 3] 3 [3 3] 4 [3.75 4] 3.5 [3 4]
Presence of artefacts	Yes = 6, no = 2	Yes = 0, no = 8
Type of artefacts	Low SNR (*n* = 5), blurring (*n* = 5)	-

Scoring method according to Bae et al [19] Data are presented as unacceptable [1] to good [3] for the depiction of fissures, unacceptable [1] to excellent [5] for the depiction of vessels and bronchi, unacceptable [1] to minimum of noise/artefacts [5] for artefacts and unacceptable [1] to superior [5] for overall acceptability

*ZTE* zero-echo time, *PROPELLER* periodically overlapping, *ParalLEL* lines with enhanced reconstruction, *SNR* signal-to-noise ratio

### Quantitative analyses

**Fig. 3:**
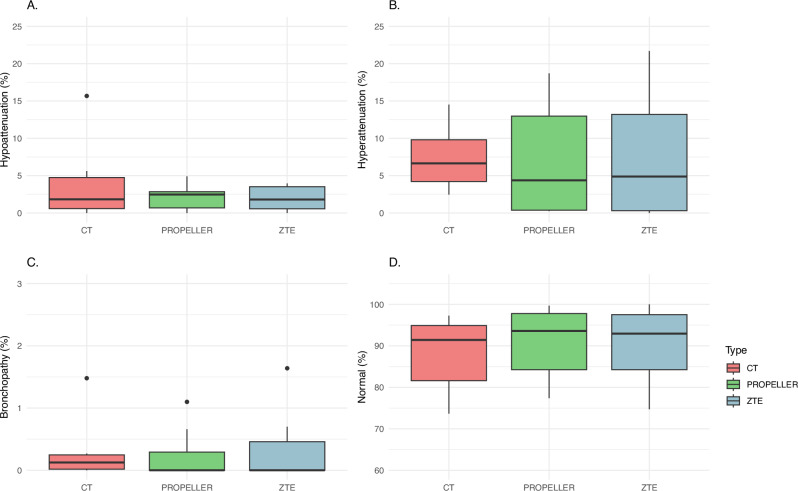
Boxplots comparing the PRAGMA-BPD score from chest CT-scan and the MERGE score for the PROPELLER and ZTE MRI sequences across all patients. **A** Percentage hypoattenuation, **B** percentage of hyperattenuation, **C** percentage of bronchopathy, and **D** percentage of normal lung tissue

MERGE scoring results are summarised in Fig. 3. No significant differences were found between CT, PROPELLER, and ZTE acquisition for the main quantitative metrics. Both MRI sequences performed similarly in classifying pathological tissue types. Across all patients, a median 10% of lung tissue was classified as diseased. In the control subject, 5.3% of lung tissue was classified as diseased (0.9% hypointense regions, 4.4% hyperintense regions, 0% bronchopathy) with hyperintense regions mainly located in the dorsal lung, likely representing dorsal atelectasis.

**Fig. 4:**
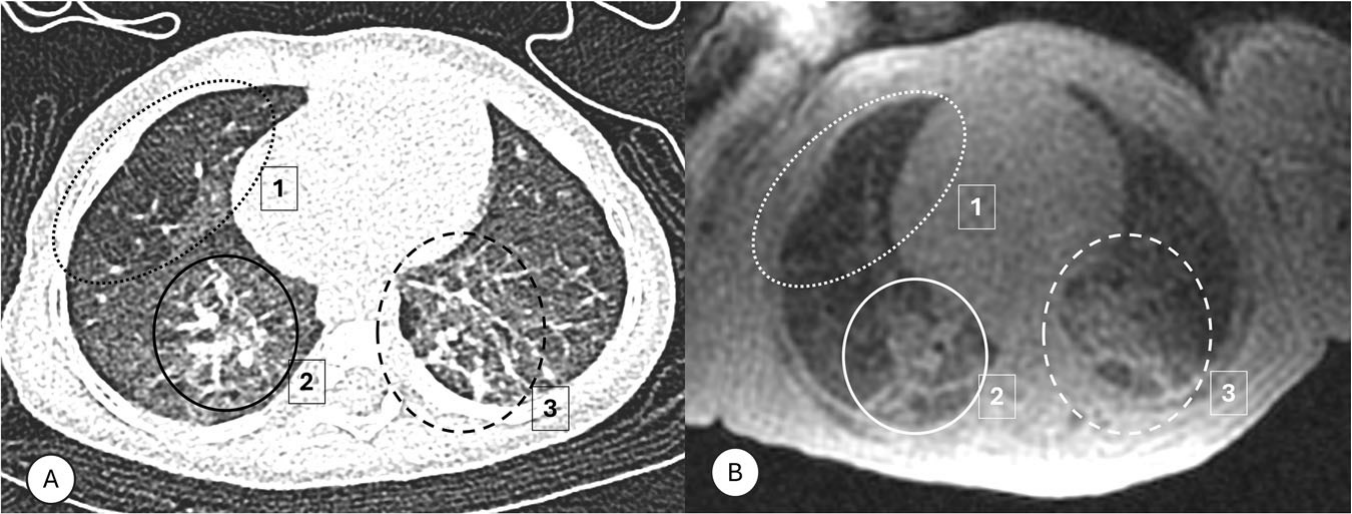
Comparison of **A** CT and **B** ZTE MRI sequence at corresponding anatomical levels in a BPD patient. Circle 1 (dotted line) highlights a hypoattenuated region on the CT-scan, which is more difficult to identify on the MRI. Circle 2 (continuous line) shows fibrotic strings visible on both CT and MRI. Circle 3 (dashed line) marks a combination of hypoattenuated regions and fibrotic strings, clearly visible on both imaging modalities. Minimal progression was observed between the two scans, with a slight improvement on the CT. ZTE, Zero echo time; BPD, Bronchopulmonary dysplasia

**Fig. 5:**
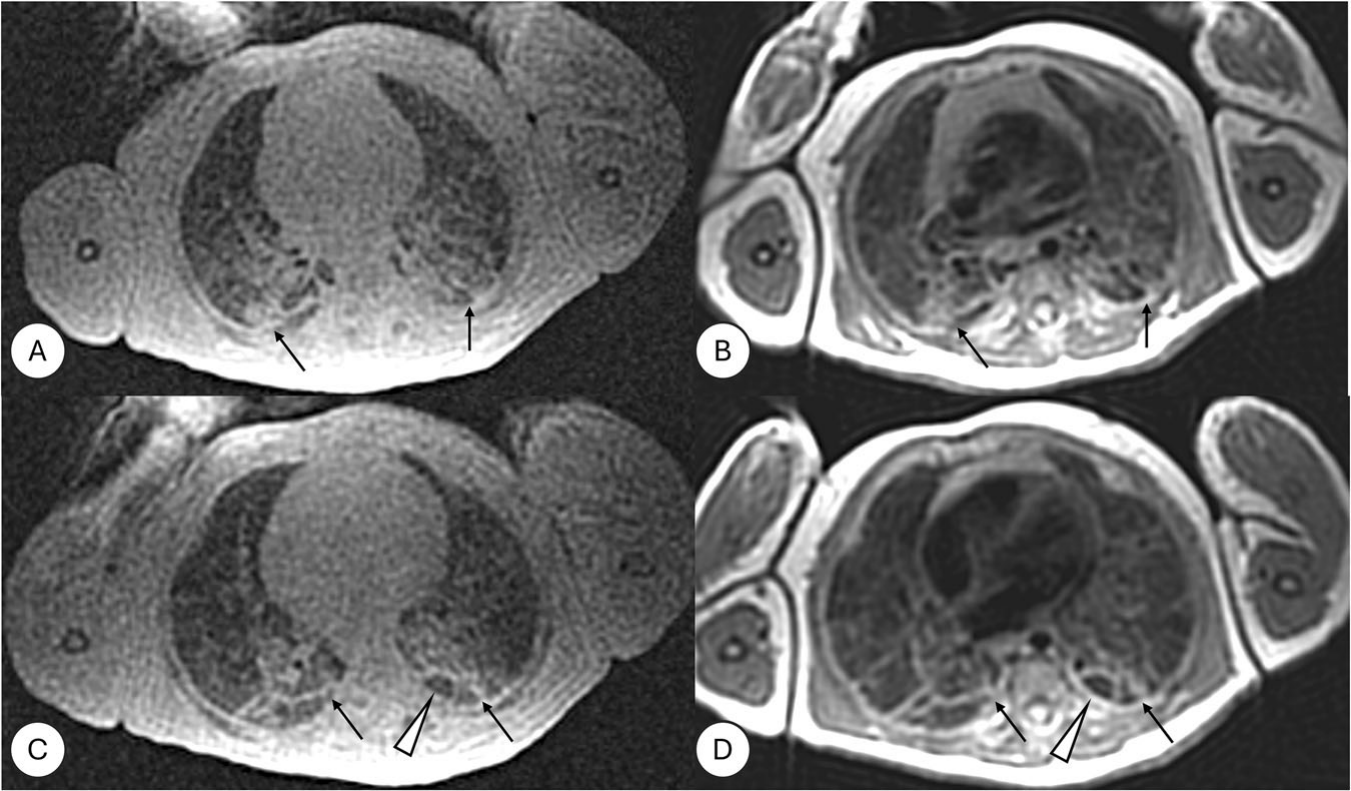
Axial PD-weighted ZTE (**A**, **C**) and axial T2-w PROPELLER (**B**, **D**) sequences of patient 1 at corresponding locations, showing hyperintense regions (atelectasis and linear opacities, arrows) and hypointense regions (arrowhead). The MERGE score showed an average of 21.7% diseased lung tissue, with hyperintense regions accounting for 16.6% of total lung volume. ZTE, zero echo time; PROPELLER, periodically rotated overlapping parallel lines with enhanced reconstruction

**Fig. 6:**
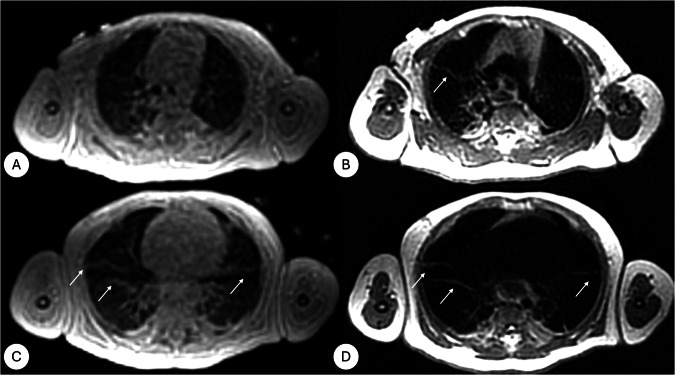
Axial ZTE (**A**, **C**) and axial T2-w PROPELLER (**B**, **D**) sequences of patient 2 at corresponding locations, showing hyperintense regions (linear opacities, arrows). Note that on ZTE images (**A**, **C**), the hyperintense regions are difficult to identify due to a low SNR ratio. The MERGE score showed an average of 14.5% diseased lung tissue, with hyperintense regions comprising 12.1% of the total lung volume. No bronchopathy was seen in this patient. ZTE, zero echo time; PROPELLER, periodically rotated overlapping parallel lines with enhanced reconstruction; SNR, signal-to-noise ratio; MERGE, the bronchopulMonary RottErdam MRI morpholoGy scorE

Comparison of MERGE score with PRAGMA-BPD CT scores showed significant correlations between PROPELLER and ZTE MRI sequences and CT for normal and hyperattenuated lung tissue (*p* < 0.05, Pearson’s *r *= 0.84-0.96). PROPELLER and ZTE were also significantly correlated with each other (*p* < 0.05, Pearson’s *r* = 0.84, 0.97) but not with CT for hypoattenuated tissue and bronchopathy (*p* > 0.2, Spearman’s *r* = –0.57–0.49. Scatter plots with correlation coefficients are available in Supplement 2. MRI and CT scans were performed more than 200 days apart, which may have contributed to variability in observed correlations. Figure 4 provides an exemplary comparison of MRI and CT images from the same patient, while Figs. 5 and 6 illustrate differences between ZTE and PROPELLER sequences in two BPD patients.

### Inter and intra scorer agreement

Supplemental Fig. 1 illustrates Bland–Altman plots of inter- and intra-scorer agreement for MRI tissue classification. Inter-scorer variability exceeded intra-scorer variability. The inter-scorer analysis demonstrated a mean bias of 5.14% for normal/healthy tissue on ZTE scans and 4.27% on PROPELLER scans. Intra-scorer agreement, assessed from repeated measurements from researcher 1, showed a mean bias of −1.9% for PROPELLER and −0.28% for ZTE.

## Discussion

This pilot study developed and tested a neonatal chest MRI protocol for assessing severe BPD-associated structural lung abnormalities using a conventional 1.5-T MRI system equipped with a dedicated neonatal chest coil. We demonstrated that chest MRI is technically feasible without anaesthesia, using the feed-and-swaddle technique. A 30-min MRI protocol including at least one PROPELLER and one ZTE sequence was successfully completed in all participants, with minimal movement artefacts observed in only one sequence from a single patient. The presence of a healthcare professional during all MRIs, providing a pacifier and sucrose, improved compliance, underscoring its importance for safety and for minimising infant stress during the procedure [24].

### T2-w PROPELLER vs PD-weighted ZTE

In terms of image quality, the T2-w PROPELLER sequence consistently provided superior tissue contrast and fewer motion artefacts. The PD-w ZTE sequence exhibited lower SNR and increased motion blurring but remained of acceptable diagnostic quality [19]. Importantly, the ZTE sequence was both faster (~ 4 min vs ~9 min for PROPELLER) and silent, which can reduce anxiety and improve compliance in neonates. While PROPELLER benefited from a higher number of averages (number of excitations = 6 vs 4 for ZTE), its longer scan time may increase the risk of motion, although our quantitative analyses did not indicate a clear difference in performance between the two sequences. Previous research showed excellent lung parenchyma visualisation on T2-w imaging and superior identification of hypointense regions on ultra-short echo time sequences in the neonatal population [13, 15, 18]. In contrast to a prior study in older BPD patients, the quality of T2w PROPELLER sequences was better compared to PD-w ZTE [25]. Together, the two sequences are complementary: T2-w PROPELLER better depicts hyperintense abnormalities (e.g. consolidations, linear opacities, bronchial wall thickening), while PD-w ZTE enhances visualisation of hypointense changes (e.g. air trapping, emphysema) and improves patient comfort due to silent scanning.

### MRI vs CT imaging

Quantitative analyses indicated that chest MRI can identify typical BPD-related lung abnormalities. The resolution achieved with ZTE (1.0–1.3 mm^3^) was comparable to previous neonatal chest MRI studies [13–15, 18]. Although not yet at the level of a standard chest CT (± 0.5 × 0.5 × 1 mm^3^), it is a step towards improved imaging without ionising radiation. Significant correlations were observed between T2-w PROPELLER and PD-w ZTE sequences for normal tissue and hyperintense tissue. However, no correlation was observed with CT for bronchopathy and hypointense regions, which are critical disease features. This discrepancy may reflect both the inherent difficulty of visualising these findings on MRI and the low percentages detected even on CT, which challenge reproducibility. Furthermore, the mean interval of ~214 days between MRI and CT scans introduced biological variability due to lung growth, recovery, or new pathology, further limiting direct comparison [26]. For instance, increased hypoattenuated tissue on CT may represent the evolution of a COPD-like phenotype, whereas hyperattenuated regions on MRI may reflect atelectasis at the time of scanning. Therefore, these correlations should be interpreted as exploratory, without implying diagnostic equivalence between the modalities.

Hyperattenuated lung tissue was the most frequent abnormality in our cohort, typically located dorsally. These might represent atelectasis [27, 28], fluid accumulation due to the infant’s position during imaging [29], or alveolar flooding [30], all of which are common in severe BPD and consistent with prior neonatal imaging studies. The single healthy control in our cohort showed markedly lower percentages of hyper- and hypointense tissue, but given the limited sample, broader comparison within a larger healthy neonatal population is needed to study how these abnormalities develop over time.

Both MRI and CT scans were conducted during free breathing, revealing a relatively low percentage of hypoattenuated tissue. This may represent an underestimation of the actual percentage of hypoattenuated tissue due to the triggering or temporal resolution of the MRI scans [17]. CT scanning in spontaneously free-breathing BPD infants allows accurate depiction of hypoattenuated regions. Therefore, future methodological improvements should include synchronisation of the MRI scans with the respiratory cycle [31] or speeding up temporal resolution using real-time MRI to overcome breathing artefacts [32].

### MRI scoring system

In contrast to prior studies [33, 34], our study provides a quantitative volumetric evaluation of structural lung abnormalities in infants with severe BPD between MR and CT images. The MERGE quantitative scoring system implemented in this study expresses abnormalities both in millilitres and as a percentage of total lung volume, offering higher measurement precision than the semi-quantitative Ochiai scheme used in earlier reports.

The new scoring system showed good intra-observer agreement, but suboptimal inter-observer agreement, likely influenced by a low percentage of diseased tissue and the novelty of the MRI-based scoring system. Although MRI and CT images were assessed by different observers to capitalise on their modality-specific expertise, a potential for reader-related bias cannot be completely excluded. Moreover, differences in scoring between ZTE and PROPELLER could have been influenced by the fixed interpretation order (ZTE first, then PROPELLER), which may have introduced a potential recall bias. While good inter-and-intra observer agreement is expected for airway visibility and hyperattenuated tissue, the identification of hypoattenuated regions remains more challenging on MRI than CT [35] due to the inherently low signal of air. Future improvement may include rater training, refined scoring system and AI-based image analysis to enhance contrast-to-noise ratio and scoring reliability, potentially enhancing detection of hypoatttenuated regions and overall inter-observer agreement [36].

### Strengths and limitations

Our study has several strengths for further development of neonatal chest MRI. First, we developed a chest MRI protocol using a conventional 1.5-T MRI system, unlike previous studies that used a specialised MRI [13]. The combination of short echo time sequences, such as PD-w ZTE and T2-w PROPELLER, was effective for detecting different types of BPD-related lung abnormalities, with the T2-w PROPELLER sequence giving the best image quality. The availability of this sequence on a generally available standard 1.5-T scanner offers the potential of broader application of chest MRI in neonates. The ZTE sequence in its latest implementation (with retrospective gating, ZTE4D) is currently available on most standard 1.5-T MRI system as a research product. The ZTE sequence with prospective gating (ZTE3D) or multiple averaging is available as a commercial product in all GE scanners. The use of silent ZTE sequences is an important innovation for neonates, reducing the likelihood of movement artefacts and improving patient comfort.

However, our study had several limitations. Firstly, the sample size was very small, with only seven severe BPD infants and 1 healthy term infant, therefore restricting generalizability. Patient inclusion was challenging due to clinical fragility, parental concerns, and transfer to peripheral centres. Secondly, the use of a dedicated neonatal chest coil, while essential for adequate SNR, is not yet standard in all institutions and may limit scalability. Thirdly, there was a significant time gap between the MRI and CT scans. This timing was chosen to align with the routinely obtained chest CT scans for infants with severe BPD at our hospital. However, matching the MRI with the routine CT would have posed additional challenges, particularly due to patient movement, which could have compromised the feasibility of performing two scans without sedation on the same day at this age. The primary CT and MRI results were assessed by different scorers, which introduces the potential for reader-related bias. Given these limitations, the results should be interpreted with caution and viewed as exploratory rather than definitive. Despite these limitations, we believe that our study provides valuable information for hospitals aiming to start MR imaging of neonates with severe prematurity-related lung disease using conventional MRI scanners.

### Future prospective

Despite employing non-Cartesian k-space acquisition schemes such as PROPELLER and ZTE, which are inherently less sensitive to motion artefacts, respiratory motion still compromised image quality in our study. A key challenge for future neonatal MRI protocol development is managing the high respiratory rate of infants (50–70 breaths per min). Technical refinements should therefore focus on improving motion compensation through retrospective or prospective respiratory gating and repeated k-space sampling to further reduce artefacts [13, 15]. Emerging real-time MRI techniques hold particular promise, offering sharp image quality in unsedated neonates without the need for external triggering [32]. The integration of AI-based reconstruction and denoising algorithms could enhance SNR and facilitate more objective, reproducible image analysis [36]. The introduction of newer ZTE implementations, such as retrospectively gated “ZTE4D” sequences, on standard 1.5-T systems may further improve robustness and accessibility for routine clinical use [37]. Finally, larger multicentre studies combining simultaneous MRI and CT acquisitions are required to validate these early findings, refine acquisition parameters, and establish standardised imaging protocols for effective clinical translation.

## Conclusions

This pilot study demonstrates that free-breathing, non-sedated neonatal chest MRI using a widely available 1.5-T scanner and a dedicated neonatal chest coil is both feasible and safe. A combined protocol including T2-w PROPELLER and PD-weighted ZTE sequences offers complementary strengths. T2-w PROPELLER delivers superior soft tissue contrast and fewer artefacts, aiding detection of hyperintense abnormalities, while PD-w ZTE provides silent, faster scanning and better visualisation of hypointense abnormalities such as air trapping. Nevertheless, MRI does not yet achieve full diagnostic equivalence with CT, as shown by the lack of correlation for bronchopathy and hypoattenuated regions and the substantial inter-observer variability. The limited sample size, single healthy control, and the large interval between MRI and CT scans further restrict the generalizability of our findings. Despite these limitations, our results provide valuable reference data for institutions seeking to implement neonatal lung MRI in severe BPD using conventional scanners. Larger, multicentre studies with simultaneous MRI–CT comparisons, extended longitudinal follow-up, and technical refinements are required to establish the clinical role of neonatal chest MRI.

## Supplementary information

ELECTRONIC SUPPLEMENTARY MATERIAL

## Abbreviations

BPD: Bronchopulmonary dysplasia
CT: Computed tomography
MERGE: The bronchopulMonary RottErdam MRI morpholoGy scorE
MRI: Magnetic resonance Imaging
PMA: Postmenstrual age
PRAGMA-BPD: Perth-Rotterdam annotated grid morphometric analysis
PROPELLER: Periodically rotated overlapping parallel lines with enhanced reconstruction
SNR: Signal-to-noise
T2-w: T2-weighted
ZTE: Zero echo time

## References

[1] Kinsella JP, Greenough A, Abman SH (2006) Bronchopulmonary dysplasia. Lancet 367:1421–1431 16650652 10.1016/S0140-6736(06)68615-7

[2] Morty RE (2018) Recent advances in the pathogenesis of BPD. Semin Perinatol 42:404–412 10.1053/j.semperi.2018.09.001 30384986

[3] Jobe AH, Bancalari E (2001) Bronchopulmonary dysplasia. Am J Respir Crit Care Med 163:1723–1729 11401896 10.1164/ajrccm.163.7.2011060

[4] Bancalari E, Jain D (2019) Bronchopulmonary dysplasia: 50 years after the original description. Neonatology 115:384–391 30974430 10.1159/000497422

[5] Ibrahim J, Bhandari V (2018) The definition of bronchopulmonary dysplasia: an evolving dilemma. Pediatr Res 84:586–588 30188504 10.1038/s41390-018-0167-9

[6] Duijts L, van Meel ER, Moschino L et al (2020) European Respiratory Society guideline on long-term management of children with bronchopulmonary dysplasia. Eur Respir J 55:1900788 10.1183/13993003.00788-2019 31558663

[7] Simpson SJ, Du Berry C, Evans DJ et al (2024) Unravelling the respiratory health path across the lifespan for survivors of preterm birth. Lancet Respir Med 12:167–180 37972623 10.1016/S2213-2600(23)00272-2

[8] Ciet P, Tiddens HA, Wielopolski PA et al (2015) Magnetic resonance imaging in children: common problems and possible solutions for lung and airways imaging. Pediatr Radiol 45:1901–1915 26342643 10.1007/s00247-015-3420-y PMC4666905

[9] Hatabu H, Ohno Y, Gefter WB et al (2020) Expanding applications of pulmonary MRI in the clinical evaluation of lung disorders: Fleischner Society Position Paper. Radiology 297:286–301 32870136 10.1148/radiol.2020201138

[10] Sodhi KS, Lee EY (2014) What all physicians should know about the potential radiation risk that computed tomography poses for paediatric patients. Acta Paediatr 103:807–811 24673144 10.1111/apa.12644

[11] Ciet P, Wielopolski P, Manniesing R et al (2014) Spirometer-controlled cine magnetic resonance imaging used to diagnose tracheobronchomalacia in paediatric patients. Eur Respir J 43:115–124 23598953 10.1183/09031936.00104512

[12] Hysinger EB, Bates AJ, Higano NS et al (2019) Ultrashort echo-time MRI for the assessment of tracheomalacia in neonates. Chest 157:595–602 10.1016/j.chest.2019.11.034 PMC7118245 31862439

[13] Hahn AD, Higano NS, Walkup LL et al (2017) Pulmonary MRI of neonates in the intensive care unit using 3D ultrashort echo time and a small footprint MRI system. J Magn Reson Imaging 45:463–471 27458992 10.1002/jmri.25394 PMC5222704

[14] Higano NS, Fleck RJ, Spielberg DR et al (2017) Quantification of neonatal lung parenchymal density via ultrashort echo time MRI with comparison to CT. J Magn Reson Imaging 46:992–1000 28160357 10.1002/jmri.25643 PMC6457694

[15] Higano NS, Hahn AD, Tkach JA et al (2017) Retrospective respiratory self-gating and removal of bulk motion in pulmonary UTE MRI of neonates and adults. Magn Reson Med 77:1284–1295 26972576 10.1002/mrm.26212 PMC5018908

[16] Higano NS, Spielberg DR, Fleck RJ et al (2018) Neonatal pulmonary magnetic resonance imaging of bronchopulmonary dysplasia predicts short-term clinical outcomes. Am J Respir Crit Care Med 198:1302–1311 29790784 10.1164/rccm.201711-2287OC PMC6290936

[17] Elders BBLJ, Tiddens HAWM, Pijnenburg MWH, Reiss IKM, Wielopolski PA, Ciet P (2022) Lung structure and function on MRI in preterm born school children with and without BPD: a feasibility study. Pediatr Pulmonol 57:2981–2991 35982507 10.1002/ppul.26119 PMC9826116

[18] Walkup LL, Tkach JA, Higano NS et al (2015) Quantitative magnetic resonance imaging of bronchopulmonary dysplasia in the neonatal intensive care unit environment. Am J Respir Crit Care Med 192:1215–1222 26186608 10.1164/rccm.201503-0552OC PMC4731620

[19] Bae K, Jeon KN, Hwang MJ et al (2019) Comparison of lung imaging using three-dimensional ultrashort echo time and zero echo time sequences: preliminary study. Eur Radiol 29:2253–2262 30547204 10.1007/s00330-018-5889-x

[20] Rosenow T, Oudraad MC, Murray CP et al (2015) PRAGMA-CF. A quantitative structural lung disease computed tomography outcome in young children with cystic fibrosis. Am J Respir Crit Care Med 191:1158–1165 25756857 10.1164/rccm.201501-0061OC

[21] van Mastrigt E, Kakar E, Ciet P et al (2017) Structural and functional ventilatory impairment in infants with severe bronchopulmonary dysplasia. Pediatr Pulmonol 52:1029–1037 28672085 10.1002/ppul.23696

[22] van Mastrigt E, Logie K, Ciet P et al (2016) Lung CT imaging in patients with bronchopulmonary dysplasia: a systematic review. Pediatr Pulmonol 51:975–986 27148803 10.1002/ppul.23446

[23] Fontijn S, Balink SJA, Bonte M et al (2023) Chest computed tomography in severe bronchopulmonary dysplasia: Comparing quantitative scoring methods. Eur J Radiol 169:111168 10.1016/j.ejrad.2023.111168 37897957

[24] Mastro KA, Flynn L, Preuster C, Summers-Gibson L, Stein MH (2019) The effects of anesthesia on the pediatric developing brain: strategies to reduce anesthesia use in pediatric MRI and nursing’s role in driving patient safety. J Perianesth Nurs 34:900–910 31196698 10.1016/j.jopan.2019.02.007

[25] Stein JM, Walkup LL, Brody AS, Fleck RJ, Woods JC (2016) Quantitative CT characterization of pediatric lung development using routine clinical imaging. Pediatr Radiol 46:1804–1812 27576458 10.1007/s00247-016-3686-8 PMC5116406

[26] McGrath-Morrow SA, Collaco JM (2019) Bronchopulmonary dysplasia: What are its links to COPD? Ther Adv Respir Dis 13:1753466619892492 31818194 10.1177/1753466619892492 PMC6904782

[27] Santos AK, Silveira J, Neves VC, Zotz TGG, Motter AA, Andreazza MG (2019) Atelectasis and lung changes in preterm neonates in the neonatal period: a blind radiological report and clinical findings. Rev Bras Ter Intensiva 31:347–353 31618354 10.5935/0103-507X.20190047 PMC7005957

[28] Dominguez MC, Alvares BR (2018) Pulmonary atelectasis in newborns with clinically treatable diseases who are on mechanical ventilation: clinical and radiological aspects. Radiol Bras 51:20–25 29559762 10.1590/0100-3984.2016.0157 PMC5846321

[29] Adams EW, Harrison MC, Counsell SJ et al (2004) Increased lung water and tissue damage in bronchopulmonary dysplasia. J Pediatr 145:503–507 15480375 10.1016/j.jpeds.2004.06.028

[30] Adams EW, Counsell SJ, Hajnal JV et al (2002) Magnetic resonance imaging of lung water content and distribution in term and preterm infants. Am J Respir Crit Care Med 166:397–402 12153978 10.1164/rccm.2104116

[31] Higano NS, Bates AJ, Gunatilaka CC et al (2022) Bronchopulmonary dysplasia from chest radiographs to magnetic resonance imaging and computed tomography: adding value. Pediatric Radiology 52:643–660 35122130 10.1007/s00247-021-05250-1 PMC8921108

[32] Hirsch FW, Frahm J, Sorge I et al (2023) Real-time MRI: a new tool of radiologic imaging in small children. Eur J Pediatrics 182:3405–3417 10.1007/s00431-023-04996-0 PMC10460313 37249681

[33] Zanette B, Schrauben EM, Munidasa S et al (2022) Clinical feasibility of structural and functional MRI in free-breathing neonates and infants. J Magn Reson Imaging 55:1696–1707 35312203 10.1002/jmri.28165

[34] Dyke JP, Voskrebenzev A, Blatt LK et al (2023) Assessment of lung ventilation of premature infants with bronchopulmonary dysplasia at 1.5 Tesla using phase-resolved functional lung magnetic resonance imaging. Pediatr Radiol 53:1076–1084 36737516 10.1007/s00247-023-05598-6

[35] Ciet P, Serra G, Bertolo S et al (2016) Assessment of CF lung disease using motion corrected PROPELLER MRI: a comparison with CT. Eur Radiol 26:780–787 26024847 10.1007/s00330-015-3850-9

[36] Papp D, Castillo T JM, Wielopolski PA et al (2023) Deep learning for improving ZTE MRI images in free breathing. Magn Reson Imaging 98:97–104 36681310 10.1016/j.mri.2023.01.019

[37] Bae K, Jeon KN, Hwang MJ et al (2020) Respiratory motion-resolved four-dimensional zero echo time (4D ZTE) lung MRI using retrospective soft gating: feasibility and image quality compared with 3D ZTE. Eur Radiol 30:5130–5138 32333146 10.1007/s00330-020-06890-x

