# Supplementary material for: Assessing the feasibility of neonatal chest MRI for bronchopulmonary dysplasia using a standard 1.5-Tesla scanner
Source: Eur Radiol. 2026 Mar 24;36(8):6399–409. doi: 10.1007/s00330-026-12452-4 (PMC13341914; doi:10.1007/s00330-026-12452-4)
Supplement: Supplementary file 1 — ELECTRONIC SUPPLEMENTARY MATERIAL [file 330_2026_12452_MOESM1_ESM.pdf]

Assessing the Feasibility of Neonatal Chest MRI for BronchoPulmonary Dysplasia Using a Standard 1.5 Tesla Scanner
ELECTRONIC SUPPLEMENTARY MATERIAL

Supplement 1

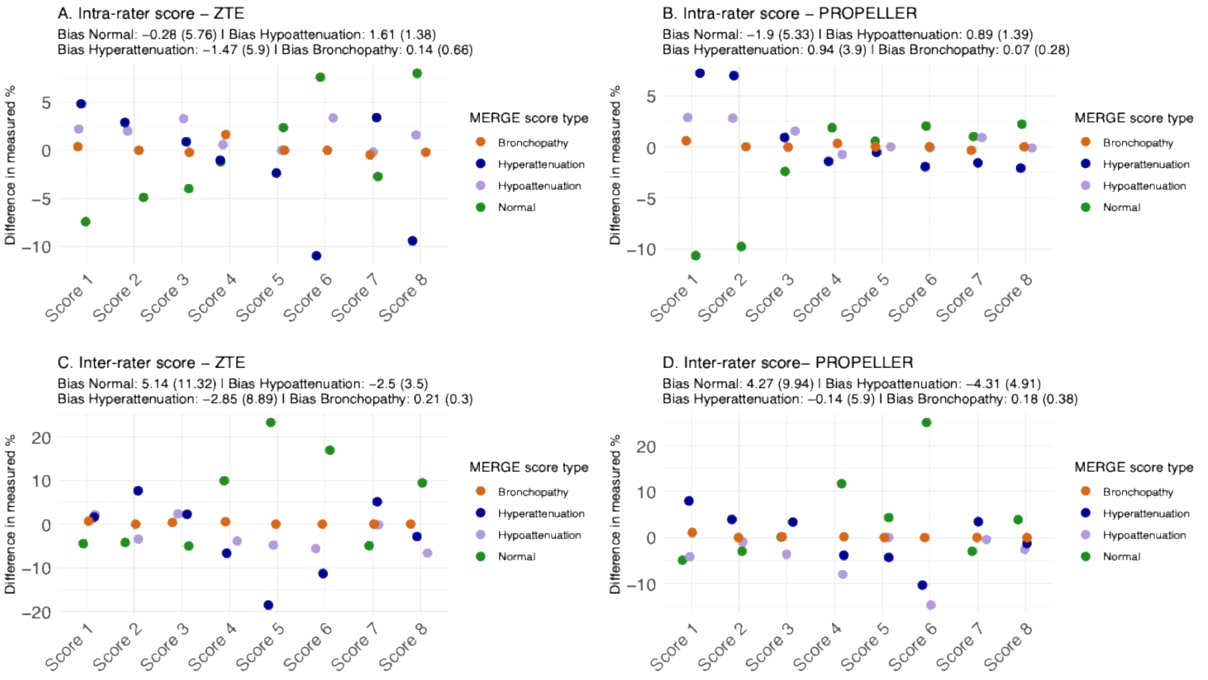

Figure S-1: Inter- and intra-rater scores for the MERGE scores based on the PROPELLER and ZTE sequences. (A) Intra-rater score for the ZTE score, showing the difference in tissue type percentages (e.g., % normal tissue by researcher 1 minus % normal tissue by researcher 2). (B) Intra-rater score agreement for the PROPELLER score. (C) Inter-rater score for the ZTE score. (D) Inter-rater score for the PROPELLER score.

Supplement 2

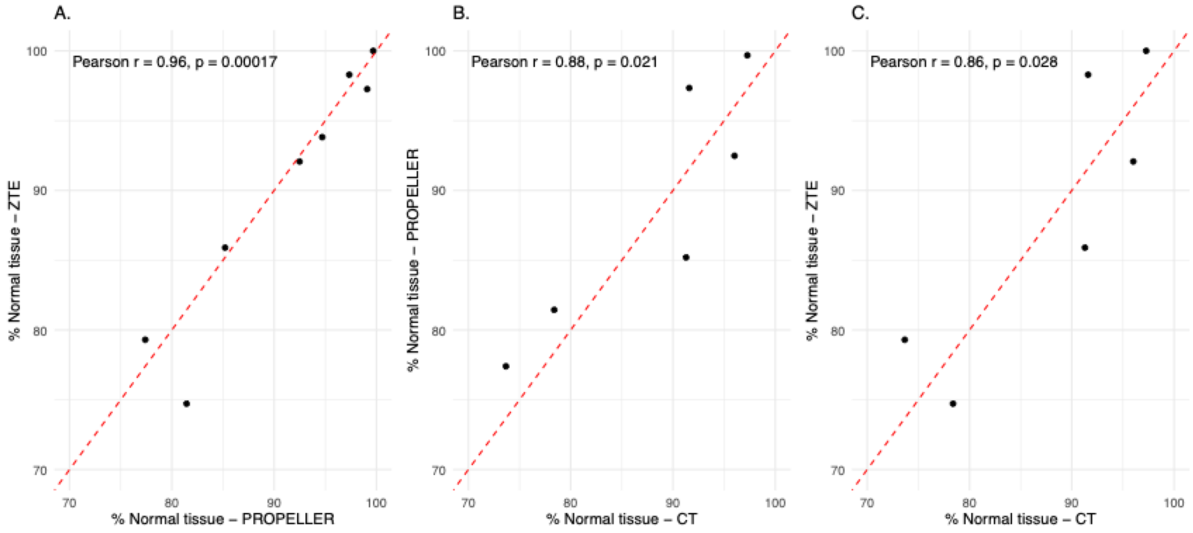

Figure S – 2.1: Scatter plot of the percentage of normal lung tissue as quantified with the PRAGMA-BPD score and the MERGE score on MRI. Red striped line at 45% indicating a perfect correlation. A. % of normal tissue MERGE score of the ZTE sequence and the MERGE score of the PROPELLER. B. % of normal tissue MERGE score of the PROPELLER sequence and the PRAGMA-BPD score on the CT sequence. C. % of normal tissue MERGE score of the ZTE sequence and the PRAGMA-BPD score on the CT sequence.

Pearson's correlation coefficient was used to calculate the statistics, based on the assumption of normally distributed data (Shapiro-Wilk test >0.05).

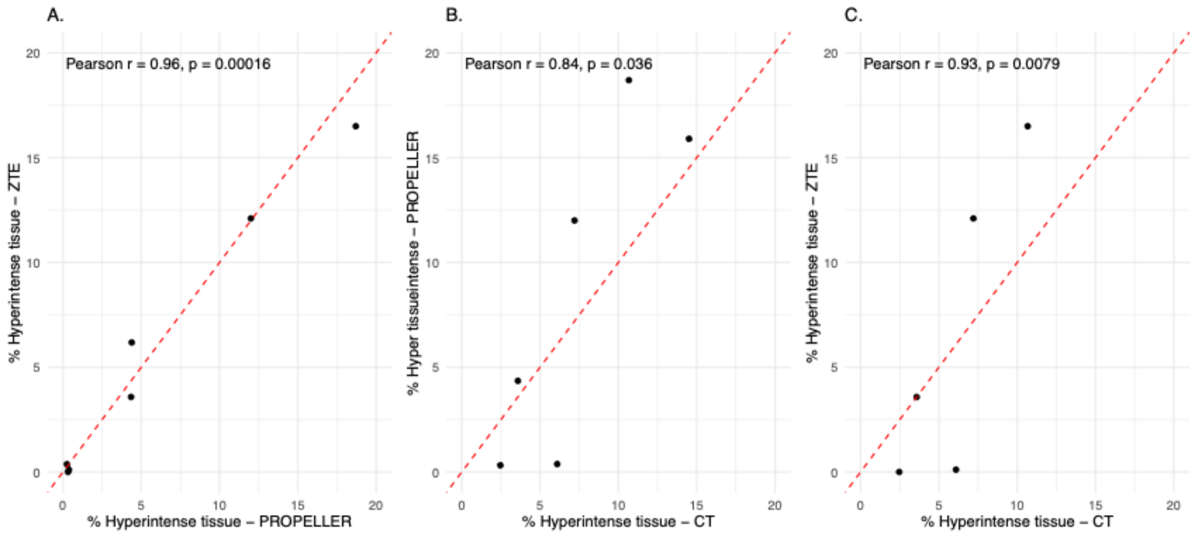

Figure S – 2.2: Scatter plot of the percentage of hyperintense lung tissue as quantified with the PRAGMA-BPD score and the MERGE score on MRI. Red striped line at 45% indicating a perfect correlation A. % of hyperintense tissue MERGE score of the ZTE sequence and the MERGE score of the PROPELLER. B. % of hyperintense tissue MERGE score of the PROPELLER sequence and the PRAGMA-BPD score on the CT sequence. C. % of hyperintense tissue MERGE score of the ZTE sequence and the PRAGMA-BPD score on the CT sequence.

Pearson's correlation coefficient was used to calculate the statistics, based on the assumption of normally distributed data (Shapiro-Wilk test >0.05).

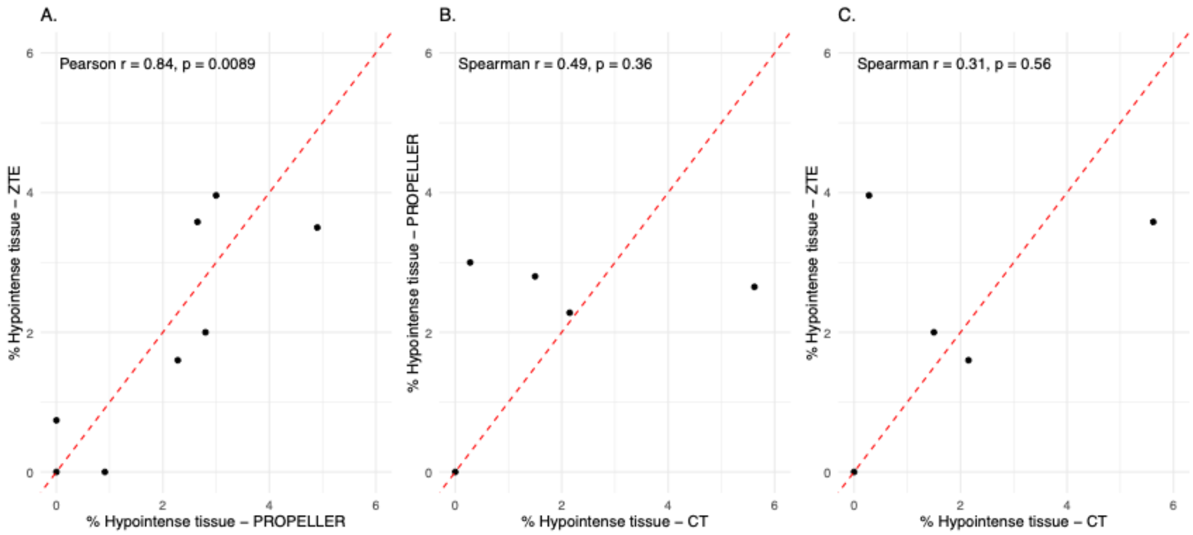

Figure S – 2.3: Scatter plot of the percentage of hypointense lung tissue as quantified with the PRAGMA-BPD score and the MERGE score on MRI. Red striped line at 45% indicating a perfect correlation A. % of hypointense tissue MERGE score of the ZTE sequence and the MERGE score of the PROPELLER. B. % of hypointense tissue MERGE score of the PROPELLER sequence and the PRAGMA-BPD score on the CT sequence. C. % of hypointense tissue MERGE score of the ZTE sequence and the PRAGMA-BPD score on the CT sequence.

Pearson's or Spearmans correlation coefficient was used to assess statistical relationships, based on the assumption of data normality by the Shapiro-Wilk test ($p>0.05$ for normal distribution, $p<0.05$ for non-normal distribution).

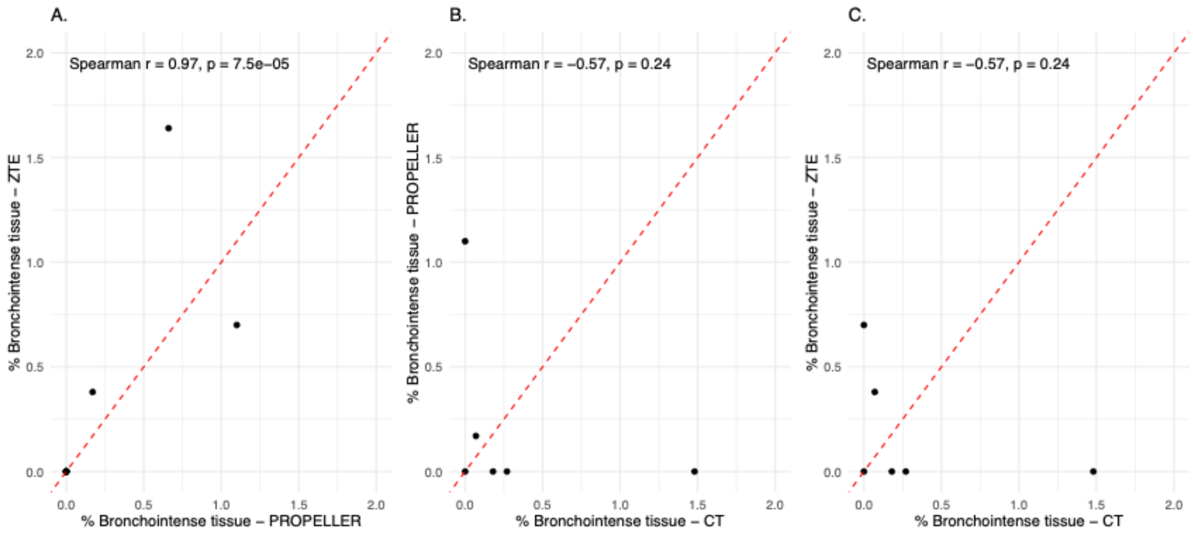

Figure S – 2.4: Scatter plot of the percentage of broncho-intense lung tissue as quantified with the PRAGMA-BPD score and the MERGE score on MRI. Red striped line at 45% indicating a perfect correlation A. % of broncho-intense tissue MERGE score of the ZTE sequence and the MERGE score of the PROPELLER. B. % of broncho-intense tissue MERGE score of the PROPELLER sequence and the PRAGMA-BPD score on the CT sequence. C. % of broncho-intense tissue MERGE score of the ZTE sequence and the PRAGMA-BPD score on the CT sequence.

Pearson's or Spearmans correlation coefficient was used to assess statistical relationships, based on the assumption of data normality by the Shapiro-Wilk test (p>0.05 for normal distribution, p<0.05 for non-normal distribution).
